# Case report of a criminal dismemberment in Northeast Brazil

**DOI:** 10.1080/20961790.2022.2055828

**Published:** 2022-05-12

**Authors:** Evelyne Pessoa Soriano, Maria do Socorro Dantas de Araújo, Francisca de Assis Nascimento Pereira, Francisca Divina Silveira de Melo, Cristiane Helena da Silva Barbosa Freire, Marcus Vitor Diniz de Carvalho

**Affiliations:** a Medico-legal Institute of João Pessoa, Paraíba, Brazil; b Faculty of Dentistry (FOP), Center for Studies in Forensic Anthropology (CEAF), University of Pernambuco (UPE), Recife, Brazil

**Keywords:** Forensic sciences, forensic anthropology, dismemberment, human identification

## Abstract

Several decomposed body parts were received for examination by the Forensic Anthropology section of the Medico-legal Institute of João Pessoa, Paraíba, Brazil. The portions of the lower and upper limbs, ribs, vertebrae, and a skull were thoroughly examined. The biological profile indicated a male individual with an estimated age range between 23 and 57 years and a mean age of 35.2 years (SD = 9.4; phase IV, Suchey-Brooks). The skeleton showed injuries caused by sharp force and sharp-blunt force trauma that affected all body segments. Macroscopically, the lesions are mainly in the diaphyseal segments of the long bones, sacrum, pelvis, mandible, maxilla, scapulae, sternum, vertebrae, the distal epiphysis of the left fibula, and the distal epiphysis of the left tibia displayed characteristics compatible with injuries produced perimortem. It was not possible to determine the cause of death. DNA analysis resulted in a positive identification. Because of common difficulties faced in forensic practice, it is often not possible for forensic anthropologists to go to the crime scene, X-ray or body scanners are frequently unavailable, and the victim’s medical and/or dental records are sometimes absent. These difficulties make identification ultimately depend on genetic analysis, which is more time-consuming than other identification methods. Despite this, bone fragment examination in dismemberment cases is a complex task. Forensic Anthropology can shed light on cases involving the identification of dismembered remains, which are challenging because of the number of traumatic injuries, as well as different injury patterns, on bones.

## Introduction

Violence has existed since the very most ancient societies and is one of the most studied subjects in forensic literature, presenting itself differently among various countries and cultures [1]. The intentional removal of body segments, referred to as dismemberment [2,3], is a relatively rare offense [1,3–5] when all kinds of violent deaths are considered. Nowadays, most dismemberment cases are related to homicides.

In Brazil, the last official report on violence [6] showed that in 2017 alone there were over 65 000 homicides in the country. The Northeast region, from where the case presented here comes from, has high rates of homicides with numbers ranging from 64 to over 220 per 100 000 inhabitants. Because of this background, the Forensic Anthropology Sections of the Brazilian Medico-Legal Institutes receive many cases per year.

As described by Trindade Filho and Machado [7], the most common motivations for dismemberment following a homicide in Brazil are (i) to facilitate the transport and disposal of the body (defensive dismemberment), (ii) outrage, as in cases of lynching someone that committed a serious crime such as rape (aggressive dismemberment), and (iii) criminal organisations using it as a demonstration of power, with several victims tortured, shot, or stabbed, and later subjected to decapitations and deprivement of other parts of their bodies.

Here, we present a case of dismemberment that occurred in 2019 in the city of João Pessoa, Brazil, and describe some of the difficulties facing forensic practice.

## Case report

**Figure 1.**
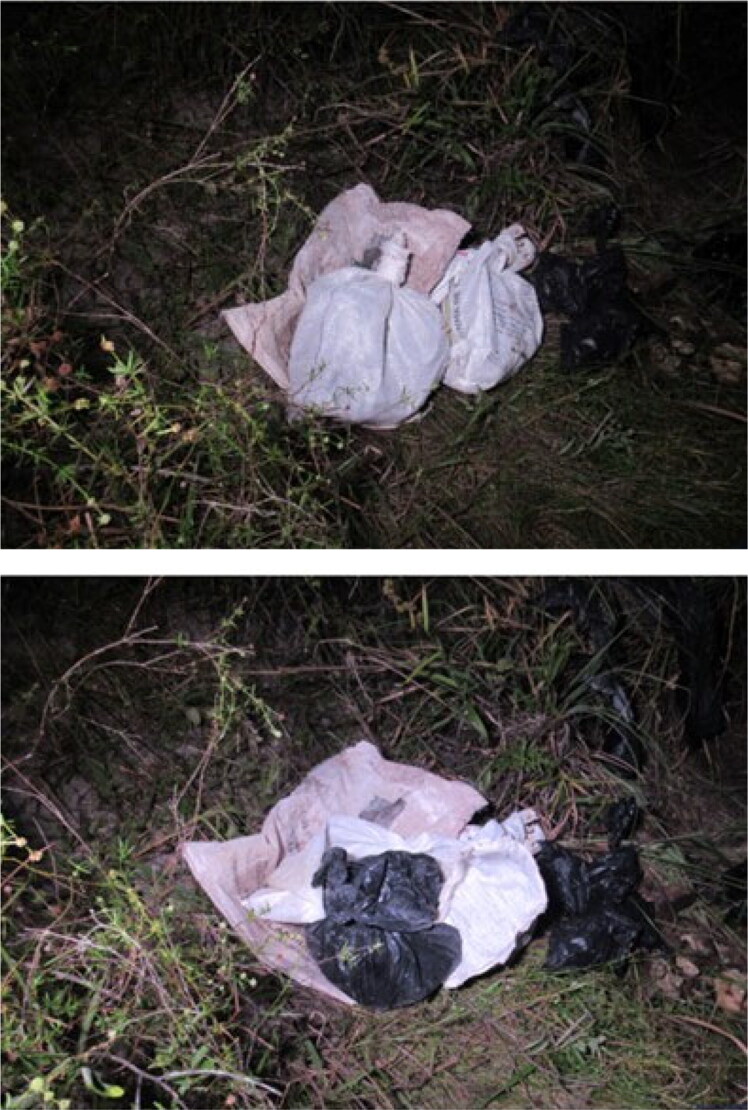
Plastic bags in which the body parts were found at the crime scene.

**Figure 2.**
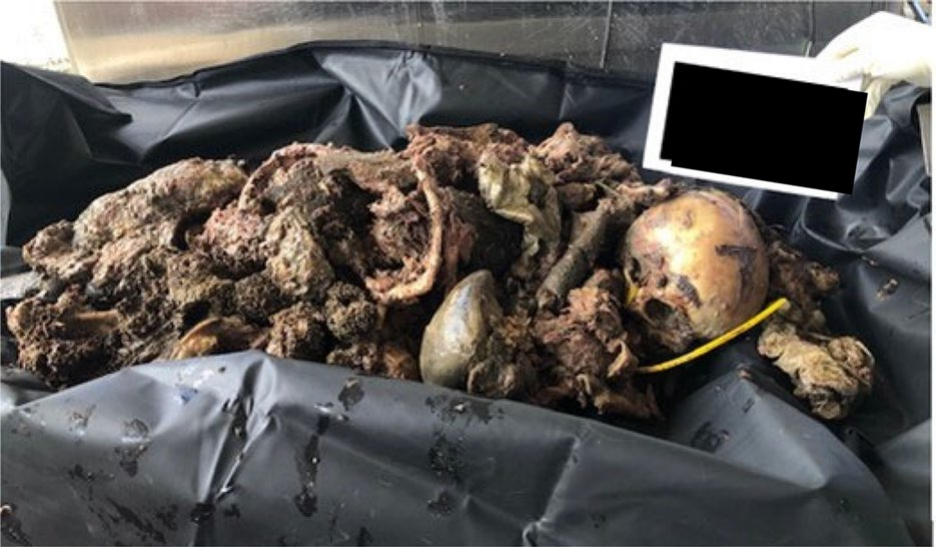
Decomposed body parts received at the Medico-Legal Institute.

Decomposed body parts were found in an open area of the metropolitan region of João Pessoa, the capital of the state of Paraíba, Northeastern Brazil. Several body parts with decomposed soft tissues were inside closed, untorn plastic bags that were spread over a forest area (Figure 1). These were recovered from the crime scene, then sent to the Forensic Anthropology Section of the Medico-Legal Institute of João Pessoa (Figure 2). The upper and lower limbs, as well as the thorax, were segmented. There was a large amount of putrefying soft tissue in the sectioned structures.

A woman contacted the Forensic Anthropology Section claiming the examined dead body was her son. According to her, he had been missing for about 15 days. As she did not have any medical or dental records of her missing son, she was referred to the Forensic Genetics Section to provide genetic material to be compared with the sample collected from the examined body. DNA analysis was then carried out using a fragment of the right femur, which resulted in a positive identification.

The family properly signed the informed consent form agreeing with this case report. This research was approved by the Research Ethics Committee of the Federal University of Pernambuco, Brazil (Opinion No. 5254002; CAAE: 55451222.6.0000.5208). All procedures followed the guidelines and rules that regulate research involving human beings in the country.

### Examination and biological profile data

The decomposing soft tissues were removed manually, and the body segments were placed in a hydrogen peroxide solution diluted in water (1:1) to complete the cleaning process. The skeleton was then placed in the anatomical position to allow a thorough examination. After observing the gross osteological comparison and the anatomic continuity of the body parts, the experts inferred that they all belonged to the same person. The classification of the morphology of the hip bone (greater sciatic notch — “score 4”, ventral arc — “male”, subpubic concavity — “male”, ischiopubic ramus ridge — “male”, and preauricular sulcus — “absent”) [8] and the skull (nuchal crest — “score 4”, mastoid process — “score 4”, orbital margin — “score 5”, glabella-supra-orbital ridge — “score 5”, and mental eminence — “score 4”) [9] presented values consistent with a male individual. The estimated age range was between 23 and 57 years, with a mean age of 35.2 years (SD = 9.4; phase IV, Suchey-Brooks). Both clavicles presented a complete fusion of medial epiphysis, suggesting an estimated age of over 29 years [10].

Metrical and morphological information from the skull showed different patterns for ancestry estimation. AncesTrees and HefneR, two free-to-use statistical programs available on the Osteomics website [11], were used for ancestry evaluation. While metrical analysis (AncesTrees) resulted in a 93.8% probability that the bones belonged to a person of North Asian ancestry, the morphological analysis (HefneR) resulted in African ancestry with a probability of 68.8%. These are typical findings when working with individuals belonging to miscegenated populations, like the Brazilian one.

### Trauma analysis

**Figure 3.**
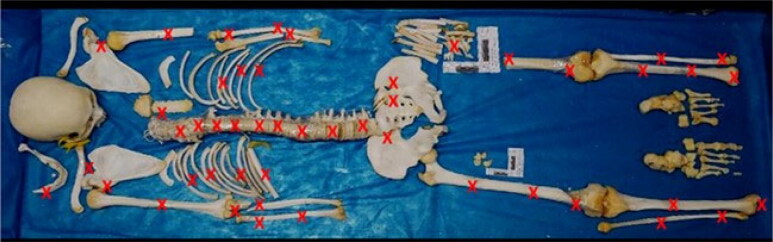
Illustration of the skeleton showing the locations of the injuries.

Several traumatic injuries on all skeleton segments (Figure 3), except the neurocranium, were observed at autopsy. These injuries were mainly caused by sharp force and sharp-blunt force trauma. No signs of animal bites were seen on the human remains analysed.

**Figure 4.**
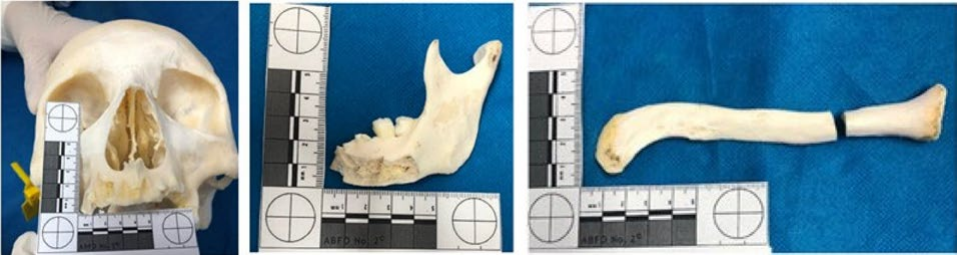
Sharp force and blunt force trauma on the maxilla, mandible, and clavicle.

**Figure 5.**
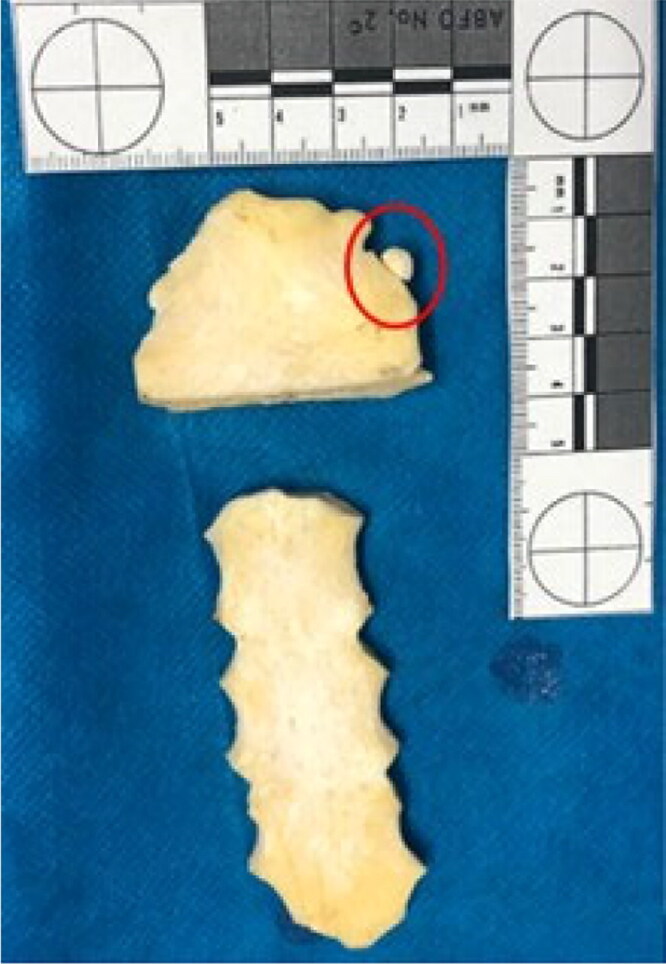
Sternum showing complete section with loss of the lower third of the manubrium. A rare anatomical variation was also seen at this site, a so-called suprasternal or episternal bone (see the red circle).

Injuries were seen on the maxilla and mandible, as well as the clavicle (Figure 4). The manubrium showed a complete section with a loss of its lower third. There was also an interesting and rare anatomical variation, a so-called suprasternal or episternal bone (Figure 5). This finding occurs in 1.5% to 4.1% of populations [12,13] and can be very useful for human identification.

**Figure 6.**
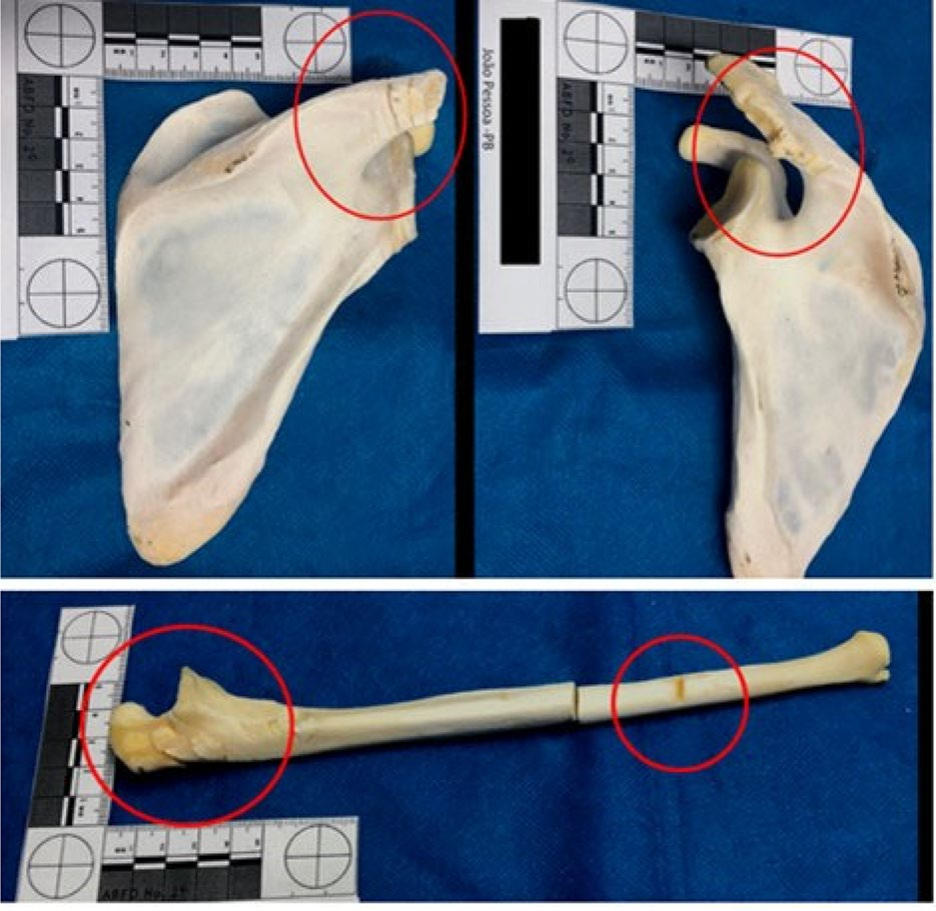
Several false starts with eversion of the bone margins on both scapulas and the proximal epiphysis of the left ulna (see the red circles).

**Figure 7.**
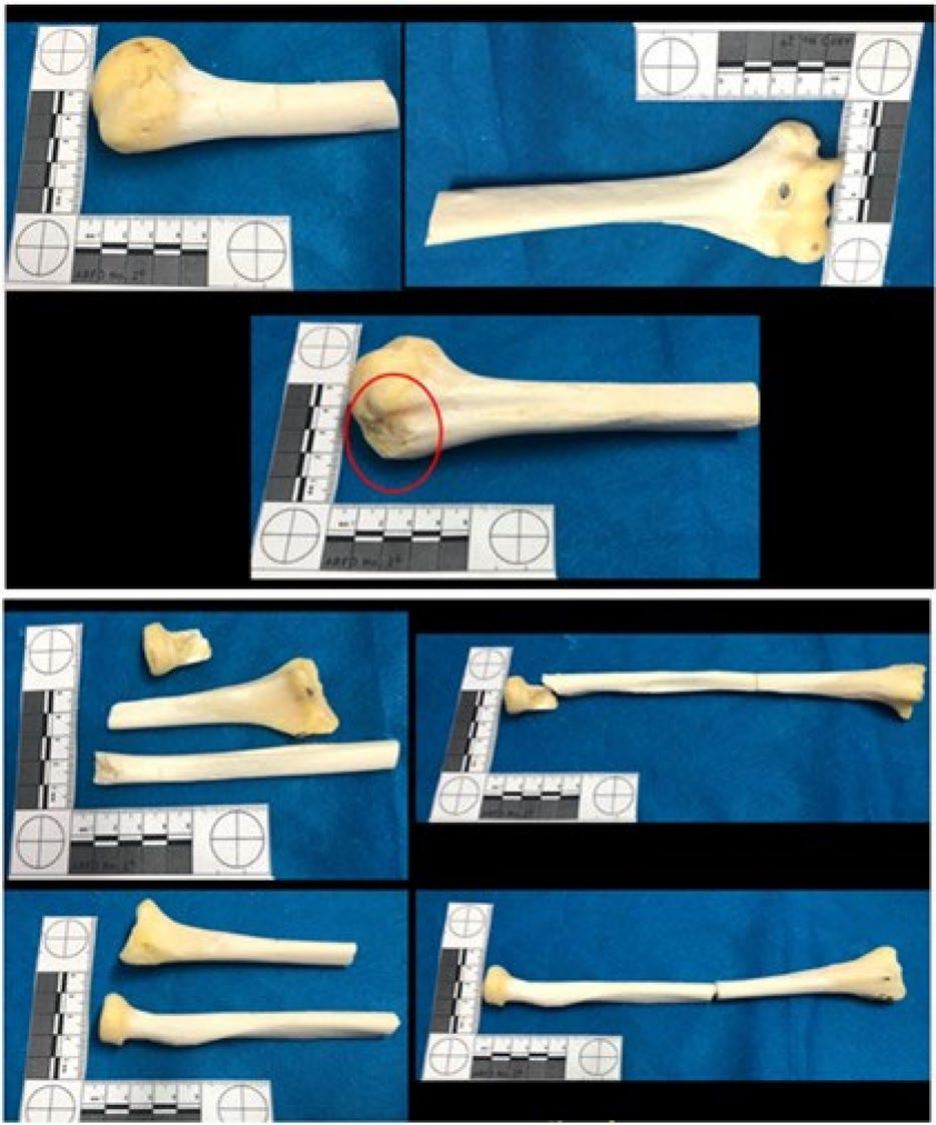
Sharp and blunt force trauma on the humerus and radius.

Both scapulas and the proximal epiphysis of the left ulna showed several false starts on its medial side, with eversion of the bone margins. The humerus and radius showed injuries caused by blunt force and sharp force trauma (Figures 6 and 7).

**Figure 8.**
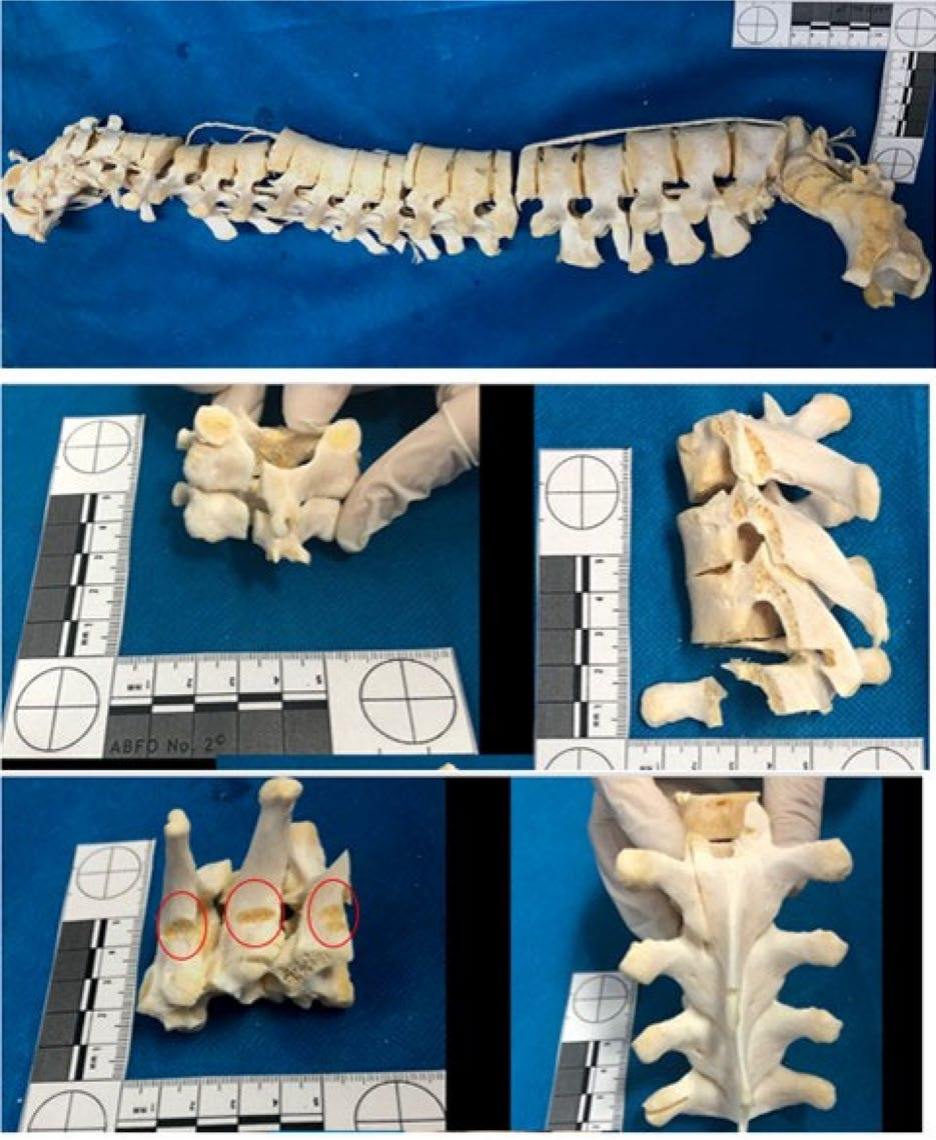
The spine mainly showed sharp force trauma on all its segments and cut impact marks (see the red circles).

**Figure 9.**
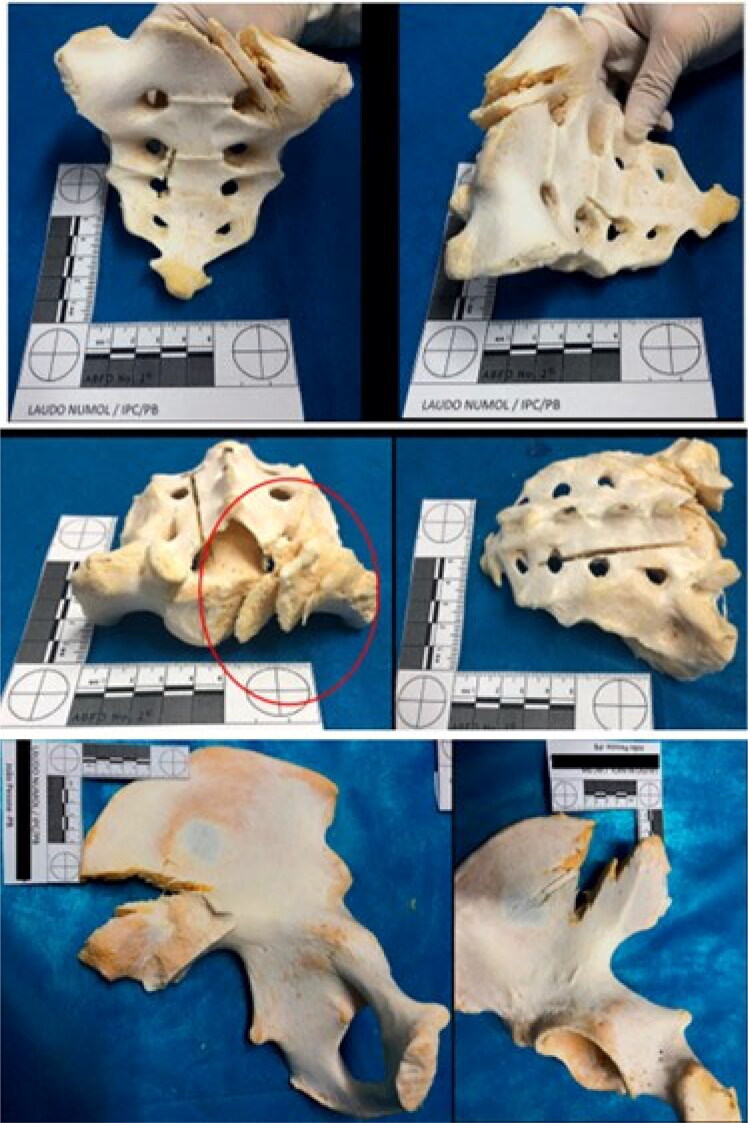
The sacrum and left ilium showed chop wounds. Cut marks, fracture lines, false starts, and hard tissue loss were observed on these bone surfaces.

The spine mainly showed sharp-blunt force trauma on all its segments and cut impact marks, as shown in Figure 8. The sacrum showed chop wounds (Figure 9), which are injuries usually caused by a heavy weapon or tool with at least one sharp cutting edge, like an axe or a machete.

**Figure 10.**
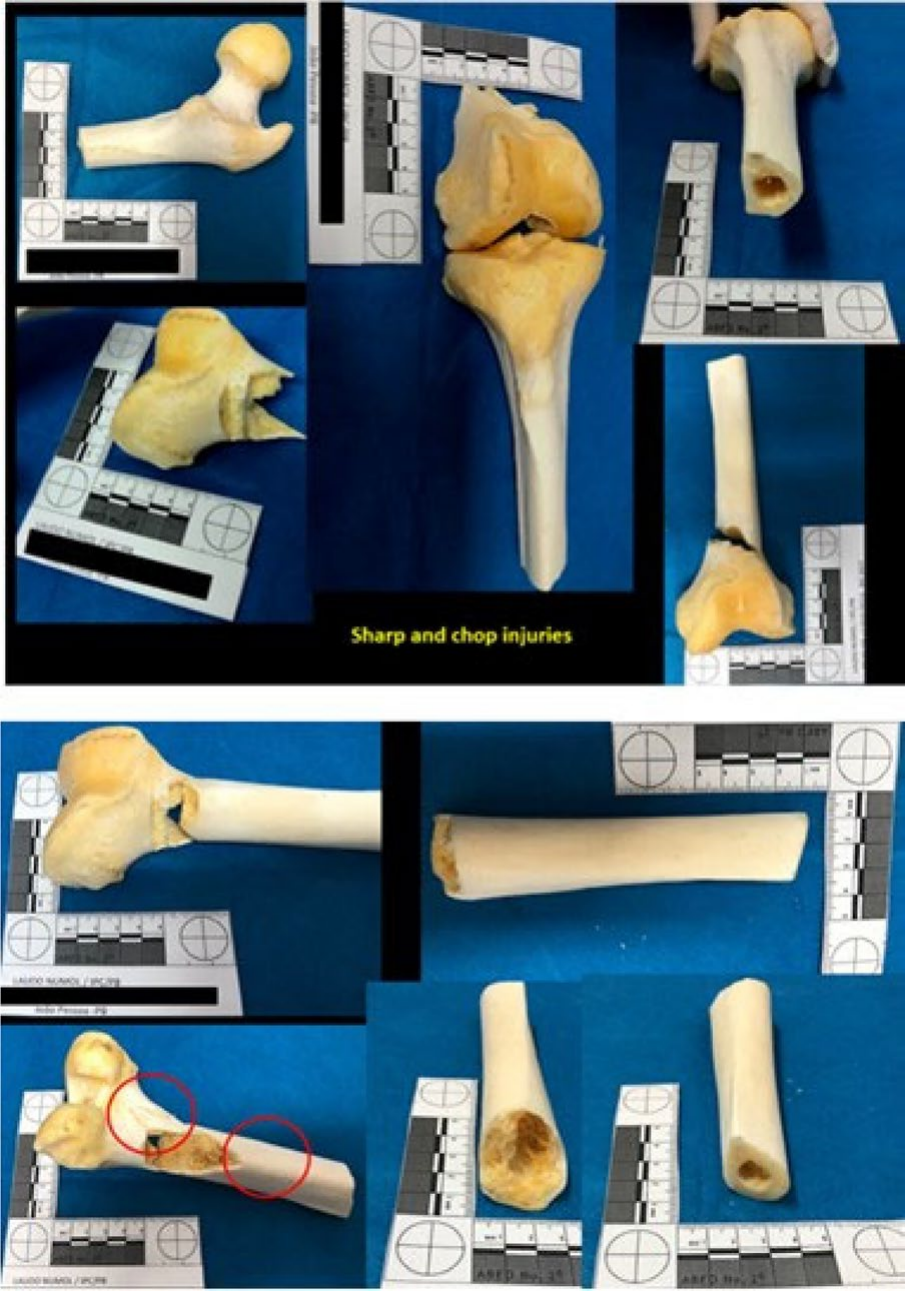
Blunt, sharp, and chop injuries at the distal epiphysis of the femurs and their diaphysis.

**Figure 11.**
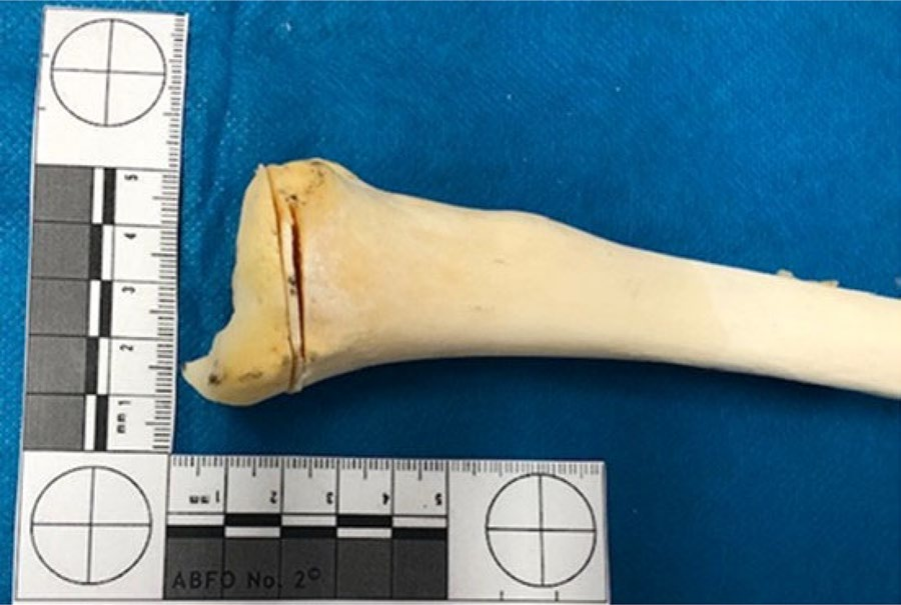
Distal epiphysis of the left tibia showed injuries consistent with false starts.

The left ilium showed chop wounds as well. Cut marks, fracture lines, and hard tissue loss were observed on the bone surface (Figure 9). The lower limbs also showed blunt, sharp, and chop injuries at the distal epiphysis of the femurs and their diaphysis (Figure 10). The distal epiphysis of the left tibia showed injuries consistent with false starts (Figure 11).

### Cause of death

No injuries were observed on the neurocranium. For the other bones, it is essential to clarify that the injuries occurring perimortem does not necessarily mean that the individual was alive at the time of the aggression but that the plastic, elastic, and anisotropic properties of the fresh bone were still present. The medico-legal examiner confirmed the dismemberment but could not unequivocally relate the presence of the observed perimortem injuries to the cause of death. The cause of death could not be conclusively determined.

## Discussion

Different types of dismemberment have been cited in the literature, with the most common being defensive, aggressive, and offensive dismemberment. Defensive involves facilitating the transport and disposal of the body or to make it challenging to identify the victim; aggressive is related to the aggressor’s impulse to dismember from fury, indignation, or emotional unrest; and offensive is when the perpetrator shows personal pleasure and gratification from inflicting pain and dismemberment, which is part of the motivation for homicide, usually driven by sexual desire, fantasy, strong anger, or sadism [1,3–5,7].

Other categories have also been cited, such as communication dismemberment, observed in some countries like Brazil in which gangs use the mutilations as a demonstration of power over a rival [1,7]. In addition, necromaniac dismemberment is when death may not necessarily result from homicide, but the aim is to use some part of the body as a symbol, trophy, or fetish [3,7].

Considering that the body parts were inside plastic bags spread over a forest area, we classified this case as a defensive dismemberment mainly perpetrated to erase proof of the crime, facilitate the disposal of the body [14], and/or make it challenging to identify the victim.

The characteristics of the injuries observed during the autopsy were mainly consistent with those caused by blunt force and sharp force trauma. They were more likely inflicted by a heavy weapon with at least one sharp cutting edge, like an axe, saw, or machete used with great energy, with sliding movements in some cases and accompanied by pressure in others. Peace et al. [15] define hacking trauma as traumatic injuries caused by chopping tools like machetes and axes, stressing that this type of injury presents characteristics of both sharp and blunt force trauma.

The response to injuries differs according to the biomechanical properties of the involved body tissue [2]. In this case, areas with false starts displaying everted margins, cut impact marks, bendings, and injuries with margins showing fracture lines were observed. These characteristics are consistent with perimortem injuries in which bone properties, primarily plastic and elastic, were still preserved.

It was not possible to determine the cause of death in this case. Determining the cause of death may be challenging, as stated by Quatrehomme and Alunni [16], because of the state of decomposition and absence of body segments. As described above, no injuries were observed on the neurocranium, and it was impossible to unequivocally relate the presence of the perimortem injuries observed on the bones to the cause of death.

One difficulty we face in forensic practice is that it is often not possible for forensic anthropologists to go to the crime scene. Consequently, some bones or other essential elements, such as teeth, may not be recovered from the crime scene. Additionally, body scanners are frequently not available in some institutes, like at ours during this case.

For victim identification, a significant difficulty lies in cases of complete skeletonization in which it is no longer possible to collect fingerprints, and the victim’s medical and/or dental records are absent. For this reason, family members are routinely referred by the police authority to provide genetic material for comparison with the information collected from the skeleton. Therefore, the identification ultimately depends on genetic analysis, which is more time-consuming than other identification methods like teeth comparisons. In the case described here, victim identification was only possible through DNA examination.

Although there are some practical difficulties and examining bone fragments in dismemberment cases is a complex task, Forensic Anthropology unquestionably contributes to cases involving the identification of dismembered remains [17,18]. These challenges are considerable because of the number of traumatic injuries on the bones, as well as the different injury patterns.
